# Insomnia and medication overuse headache: neurobiological links, reciprocal interactions, and integrated therapeutic strategies

**DOI:** 10.3389/fpain.2026.1911261

**Published:** 2026-10-16

**Authors:** Ervin Cotrik, Abouch V. Krymchantowski, Carla Jevoux, Isabela Caldas Ferreira, Leila Monteiro Auler, Maria Carolina Ferreira Fontoura Sousa, Ana Paula Marcussi

**Affiliations:** 1 Department of Medical Sciences, University of Campinas (Unicamp), Campinas, Brazil; 2 Headache Center of Rio de Janeiro, Rio de Janeiro, Brazil; 3 Universidade Estácio, Ribeirão Preto, Brazil; 4 Hospital Unimed, Volta Redonda, Rio de Janeiro, Brazil; 5 APAE/CER II de Ribeirão Preto and CAPS III AD Flávio Fortes D'andrea, Ribeirão Preto, Brazil; 6 São Leopoldo Mandic, Campinas, Brazil

**Keywords:** CBT-I, central sensitization, COMISA, insomnia, medication overuse headache, melatonin, orexin, reciprocal relationship

## Abstract

Medication overuse headache (MOH) and insomnia are not merely coincidental companions: each measurably worsens the severity and treatment response of the other. Whether this association reflects a genuine reciprocal influence, a shared constitutional vulnerability, or some combination of both carries direct practical consequences for clinical decision-making. This narrative review synthesizes evidence from Mendelian randomization genetics, polysomnographic cohort studies, pharmacological withdrawal trials, and mechanistic neurobiology, based on a search of PubMed, Embase, and Google Scholar through 20 June 2026. Genetic analyses are compatible with a directional association between insomnia liability and migraine risk, although causation is not established and the instruments capture migraine broadly rather than the MOH phenotype, a limitation that applies to all causal inferences in this review. In the other direction, opioids, barbiturates, and triptans each disrupt sleep architecture through mechanisms specific to their molecular targets, and questionnaire-based withdrawal studies consistently report parallel improvements in sleep quality and headache frequency. Three neurobiological substrates appear centrally involved: orexinergic dysregulation, melatonin suppression, and glial neuroinflammation. Together, they may sustain a self-reinforcing cycle that neither detoxification alone nor sleep treatment alone can fully dismantle. The COMISA phenotype (comorbid insomnia and obstructive sleep apnea) and concurrent psychiatric illness compound treatment resistance and raise relapse rates at one year. Objective polysomnographic evidence that sleep architecture actually recovers after drug withdrawal is absent from the literature — a gap that limits the mechanistic conclusions that can be drawn. The available evidence is consistent with the hypothesis that concurrent medication withdrawal, CBT-I, preventive pharmacotherapy — including anti-CGRP monoclonal antibodies — and CPAP where indicated may yield better outcomes than sequential management, but this hypothesis has not been tested in a randomized controlled trial. Designing that trial is the most pressing research need in this field.

## Introduction

There is a clinical pattern that recurs reliably in headache centers, and it goes largely unremarked. A patient arrives with years of daily or near-daily pain and an equally long record of escalating analgesic use. At some point in the consultation — often late, often in passing — they mention that sleep has not been satisfactory for roughly the same period. The usual response is to assign the sleep problem to the pain: fix the headache, and the rest will follow. The evidence reviewed here suggests that response is systematically wrong.

Migraine is among the most disabling neurological conditions known, affecting roughly 1.1 billion people and ranking second globally among causes of years lived with disability (1). Most patients experience the disorder episodically — attacks are severe but resolve, and headache-free days are the norm. A subset, however, follows a different course: attacks become more frequent, the headache-free baseline erodes, and eventually the diagnosis shifts to chronic migraine, which the ICHD-3 defines as headache on 15 or more days per month for at least 3 months, with at least 8 days fulfilling migraine criteria (2).

Under ICHD-3 criteria, MOH requires a pre-existing primary headache disorder, headache on 15 or more days per month, and overuse of acute medication for more than three months: triptans, ergots, opioids, or combination analgesics on 10 or more days per month, or simple analgesics on 15 or more days per month (3). In the general population it affects roughly 1%–2.5% of adults; within specialized headache centers, it accounts for approximately 30%–50% of patients presenting with chronic daily headache, with some tertiary cohorts reporting rates approaching 70% when broader chronic daily headache criteria are applied rather than strict ICHD-3 MOH (2, 4). Women outnumber men by a factor of roughly 3 to 4 (3).

Sleep complaints are nearly universal in this population, though prevalence data specific to MOH cohorts are surprisingly sparse. The oft-cited figure of 67.7% (5) derives from a case-control study of chronic headache broadly defined, not MOH specifically, in which insomnia was identified by structured sleep interview (5); in cohorts composed specifically of MOH patients, rates assessed with validated insomnia instruments range from roughly 38% to 60% (6, 7); direct comparison across studies is limited by heterogeneity in instruments, thresholds, and MOH diagnostic criteria. Comorbid depression or anxiety is present in approximately 40%–50% of cases (4). When disordered sleep and MOH coexist, both pain severity and treatment failure rates increase, and one-year relapse after successful detoxification runs between 20% and 40% (3, 8). Understanding why requires examining the directionality of the relationship, and that is where this review begins.

## Methods of narrative review

This article is a narrative review. It is not a systematic review or scoping review, and no formal protocol was pre-specified or registered. We conducted a search of PubMed (207 records) and Embase (670 records), both searched from inception to 20 June 2026, with searches executed on 16 July 2026. All 877 records were exported for deduplication and screening. Google Scholar was used only for supplementary citation tracking, restricted to the first 100 results per supplementary search sorted by relevance, to identify highly cited articles not indexed in PubMed or Embase; these were evaluated separately and did not contribute to the deduplicated record pool. After deduplication, the 877 exported records yielded 730 unique records, which were screened by title and abstract against the eligibility criteria below; records judged potentially relevant were examined in full text, and those meeting the criteria were retained for the narrative synthesis. The primary Boolean string, applied in MeSH and free-text combinations, was: (“medication overuse headache” OR “analgesic rebound headache”) AND (“sleep disorders” OR “insomnia” OR “sleep apnea” OR “orexin” OR “hypocretin” OR “melatonin” OR “circadian”). Supplementary searches combined “Mendelian randomization” OR “CBT-I” OR “dual orexin receptor antagonist” OR “CGRP” with “headache” and “sleep” — used to capture mechanistic and therapeutic evidence, not to estimate insomnia prevalence in MOH. Priority was given to randomized controlled trials, systematic reviews, and meta-analyses; relevant prospective cohort studies, mechanistic investigations in humans and validated animal models, and seminal observational studies were included when higher-level evidence was unavailable. We included English-language peer-reviewed original research and reviews published through 20 June 2026. Editorials, commentaries, and letters to the editor; conference abstracts and other non-peer-reviewed meeting material; book chapters; preprints (including bioRxiv and medRxiv); case reports unless mechanistically informative; non-English publications (none appeared among the sources cited); and duplicate or superseded reports were excluded. Beyond the study types above, eligible sources also included genetic (including Mendelian randomization) analyses and randomized trials in insomnia or migraine populations; in every case, eligibility required direct topical relevance to MOH, insomnia, orexin biology, circadian rhythms, CBT-I, DORAs, or anti-CGRP therapies. Source selection was carried out by the first author and reviewed by the senior author. No citation chasing (forward or backward) was performed systematically; trial registries (ClinicalTrials.gov, WHO ICTRP) were not searched. These are acknowledged as limitations of the search strategy. Randomized controlled trials were assessed for allocation concealment and blinding; observational studies were appraised for selection bias and confounding. Thirty-six sources informed the final synthesis. Evidence was appraised narratively, without application of a formal quality-grading framework such as GRADE. GRADE rates the certainty of evidence for comparative clinical outcomes across a body of trials addressing a defined question; no body of trials comparing integrated against sequential management of MOH with comorbid insomnia exists, and the central observation of this review is that absence. The objective of this review is therefore not to synthesize evidence of efficacy but to map the shared pathophysiological landscape and identify translational gaps that define the next research questions. This approach carries inherent limitations, including selection bias, the absence of quantitative pooling, and the non-exhaustive nature of narrative review; these are acknowledged in the limitations section.

## Putative shared pathophysiology: the neurobiology of sleep and pain

Clinical practice tends to treat sleep and pain as separate problems. The neurobiology does not support that separation. The hypothalamus, brainstem, thalamus, and cortex are embedded in both systems simultaneously, and perturbation in either direction propagates readily into the other. This anatomical overlap is probably the simplest explanation for why single-target interventions so often produce incomplete or transient responses in patients who carry both conditions (see Table 1 and Figure 1).

**Table 1:** Proposed shared neurobiological substrates linking insomnia and medication-overuse headache (MOH), and their hypothesized interactions.

Shared substrate	Role in insomnia	Role in MOH	Hypothesized interaction (how each feeds the other)	Evidence base & provenance
Orexinergic (hypothalamic) dysregulation	↑ arousal/wakefulness; dysregulated orexin signalling fragments sleep and reduces sleep continuity.	CSF orexin-A elevated in MOH (*P* < 0.000001) and, to a lesser degree, in chronic migraine (*P* < 0.0002); correlates with monthly acute-medication intake and dependence scores.	Elevated orexinergic tone may couple insomnia-related hyperarousal to trigeminovascular pain facilitation and to drug-seeking behaviour; direction of causation undetermined.	Human, MOH-specific (Sarchielli 2008) (13).
Melatonin and circadian disruption	↑ circadian disruption; blunted nocturnal melatonin delays sleep onset and impairs consolidation.	Circadian dysregulation associated with headache chronification and attack timing; loss of nocturnal analgesic/anti-inflammatory signalling.	Reduced nocturnal melatonin may remove chronobiotic and antinociceptive signalling when pain modulation is most needed, plausibly lowering the threshold for both disorders.	Migraine/indirect and preclinical; not MOH-specific.
Neuroinflammation & central (thalamocortical) sensitization	↑ nocturnal arousal, non-restorative sleep; sleep loss raises IL-1β, TNF-α, IL-6 and glial activation.	↑ central sensitization; neuroinflammation lowers pain threshold and altered thalamocortical sensory processing sustains trigeminovascular activation.	Self-reinforcing loop: poor sleep → neuroinflammation/glial activation → central sensitization → more headache and acute-medication use → further sleep disruption.	Preclinical + non-MOH-specific human imaging; thalamocortical construct not MOH-derived.

All shared interactions are hypothesized, not established; no study has tested them prospectively in MOH with comorbid insomnia. Human, MOH-specific evidence exists only for orexinergic dysregulation (Sarchielli 2008) (13); the melatonin/circadian and neuroinflammation rows rest on migraine, chronic-pain, or preclinical data. ↑ increase; ↓ decrease.

IL-1β, interleukin-1 beta; IL-6, interleukin-6; MOH, medication-overuse headache; TNF-α, tumor necrosis factor alpha.

**Figure 1:**
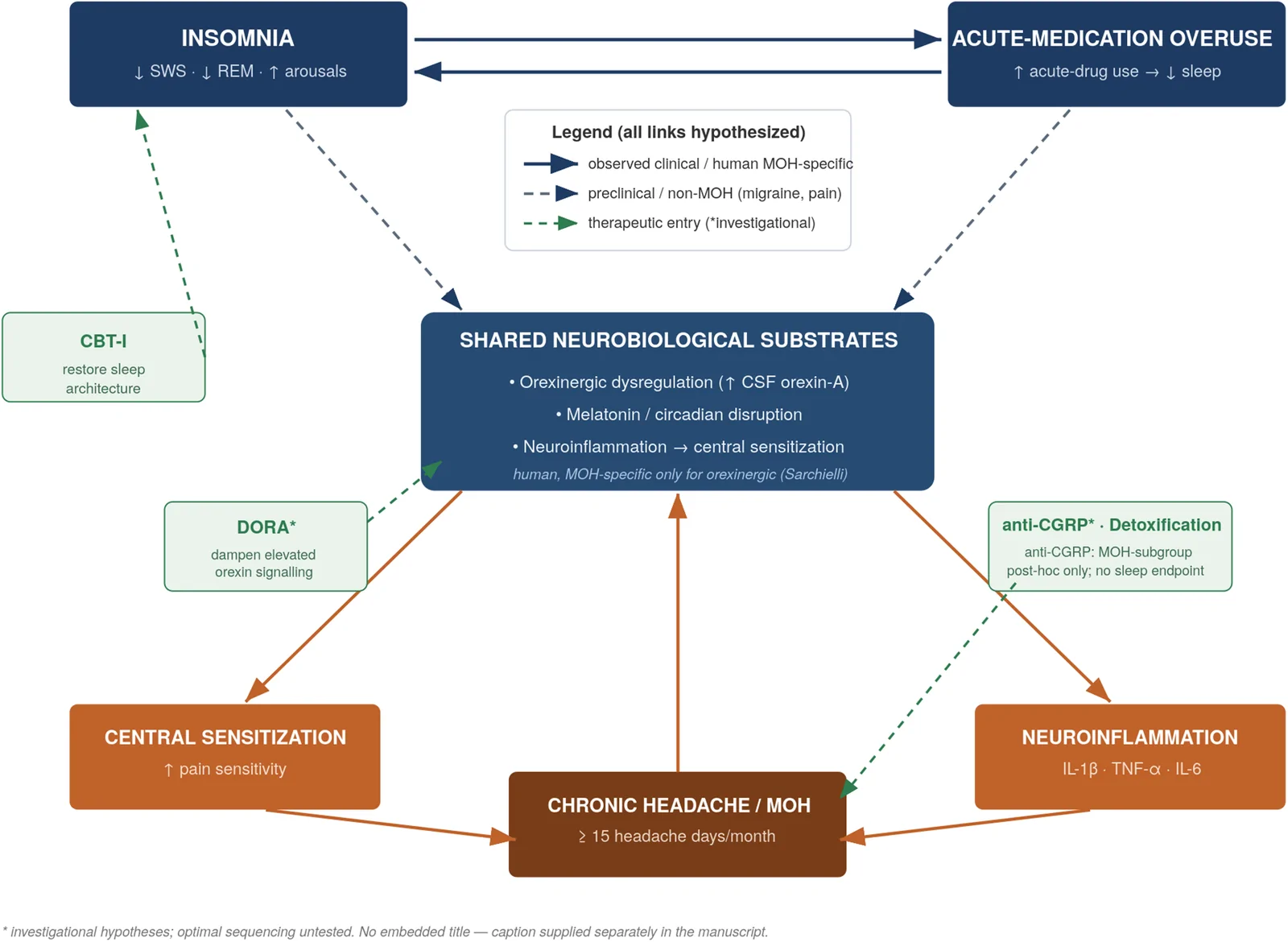
Conceptual model of the proposed reciprocal interaction between insomnia and medication-overuse headache (MOH). All depicted links are hypothesized, not established. Solid arrows denote observed clinical/reciprocal associations or human MOH-specific evidence; dashed arrows denote preclinical or non-MOH (migraine/chronic-pain) evidence. Green dashed arrows mark candidate therapeutic entry points, shown as investigational hypotheses rather than clinical recommendations, whose optimal sequencing is untested. anti-CGRP, anti-calcitonin gene-related peptide therapy; CBT-I, cognitive behavioural therapy for insomnia; DORA, dual orexin receptor antagonist; MOH, medication-overuse headache.

### The orexinergic system

Orexinergic neurons in the lateral hypothalamus project to a strikingly broad set of targets: the periaqueductal gray, the trigeminocervical complex, the rostral ventromedial medulla, the locus coeruleus, and the dorsal raphe nucleus (9, 10). That distribution is not incidental. Each of these structures plays a dual role in wakefulness maintenance and descending pain inhibition, which means a single system simultaneously regulates two functions that are both compromised in MOH.

In animal preparations, selective OX1R activation attenuates trigeminovascular nociceptive signaling; pharmacological blockade amplifies it (11). Independent human evidence for an orexinergic role in headache comes from narcolepsy, where orexin deficiency is the defining pathology and migraine prevalence is substantially elevated relative to the general population (12). The only available human, MOH-specific biomarker data, however, point in a different direction. Sarchielli et al. measured CSF orexin-A in 27 patients with chronic migraine and 30 with MOH against 20 age-matched controls, and found orexin-A significantly elevated in MOH (*p* < 0.000001) and, to a lesser degree, in chronic migraine (*p* < 0.0002), with a positive correlation between CSF orexin-A and both monthly drug intake and substance-dependence scores (13). The direction of this finding is opposite to the suppression predicted by opioid-overuse models: orexin-A is elevated, not depleted. Whether this elevation reflects a compensatory antinociceptive response to chronic pain, a stress-induced hypothalamic response, or a mechanism linked to drug-seeking behaviour (given the positive correlation with Leeds Dependence Questionnaire scores) has not been resolved — the original authors propose all three candidate explanations without adjudicating between them, and the finding predates the availability of ICHD-3-defined MOH cohorts. The practical implication for DORAs is discussed below.

### Melatonin and circadian rhythms

Melatonin is often framed as a sleep hormone, but that framing is reductive. Its nocturnal secretion by the pineal gland functions as the primary zeitgeber for the circadian system (14), and it carries documented analgesic and anti-inflammatory activity independent of its sleep-promoting effects. In patients with chronic migraine and MOH, altered circadian signaling — including possible melatonin secretion abnormalities — has been proposed as a contributing factor, though the human data specific to MOH remain indirect (15–17). Genetic data add another dimension: CK1*δ* variants, which regulate circadian clock timing, associate independently with both sleep disorders and migraine susceptibility (17), raising the possibility that some patients carry a constitutive circadian vulnerability that predisposes them to both. A single randomized trial of nightly melatonin 3 mg showed significant reduction in migraine frequency versus placebo (18). Whether the mechanism is circadian stabilization, direct analgesia, or both is not yet established.

### Trigeminovascular system and sleep architecture

Healthy sleep actively suppresses pain. During slow-wave sleep, the thalamocortical system modulates sensory relay, reducing nociceptive throughput and raising the pain threshold (19). REM is more complicated: monoaminergic descending inhibition drops to near-zero during this phase, leaving the nervous system substantially exposed to incoming nociceptive signals (20). MOH disrupts both. Polysomnographic studies document shortened total sleep time, increased nocturnal arousals, reduced SWS duration, and diminished slow-wave amplitude across this population (21). The consequence, while difficult to confirm in the absence of simultaneous pain threshold measurements, is that nociceptive signals may reach cortical processing with less attenuation than in healthy sleepers — a mechanistically coherent explanation for the morning headaches that so consistently characterize this clinical picture (21).

### Neuroinflammation and compounding cycles

Sleep deprivation is not a passive correlate of pain — it actively worsens it. Even brief periods of restricted sleep are sufficient to upregulate circulating IL-1β, TNF-α, and IL-6, drive microglial and astrocytic activation, and impair the glymphatic system's capacity to clear neuroactive metabolites from the brain interstitium (22). Chronic analgesic overuse then adds a second wave of neuroinflammatory activation through toll-like receptor 4 (TLR4) signaling on glial cells (23). These components may interact in preclinical models and indirect human data, though direct mechanistic evidence from MOH patients is limited: orexinergic dysregulation and poor sleep promote neuroinflammation, which subsequently lowers the pain threshold and increases the drive for analgesic use, perpetuating the disorder (24). Table 1 summarizes how each of these components feeds the others.

## Evidence for a reciprocal relationship

### Insomnia as a potential contributor to MOH chronification

The biological case is reasonably clear: sleep fragmentation lowers the threshold for cortical spreading depression, drives central sensitization through reduced descending inhibition and hypothalamic dysfunction, and generates hyperalgesia that compounds across nights (21). At the epidemiological level, insomnia independently predicts the transition from episodic to chronic migraine, approximately doubling or tripling the chronification probability in prospective cohort data (25). The methodological problems are equally clear: sleep data in these cohorts rely almost entirely on self-report, psychiatric comorbidity confounds interpretation at every step, and the direction of causation cannot be resolved through observational design alone — pain disrupts sleep just as reliably as sleep disrupts pain regulation.

Mendelian randomization addresses the directionality problem by using germline genetic variants as instruments for exposure. Ouyang et al. conducted a bidirectional two-sample analysis and found that genetic liability to insomnia was significantly associated with migraine risk in the discovery dataset (OR 1.02; 95% CI 1.01–1.03; *p* = 2.67 × 10⁻⁸), and this effect was confirmed in an independent validation dataset (OR 2.30; 95% CI 1.60–3.30; *p* = 5.78 × 10⁻⁶) (26). The marked difference in point estimates between the two datasets — 1.02 versus 2.30 — should caution against reading either figure as a stable population-level effect size. For the reverse direction, migraine showed a nominal effect on insomnia in the discovery dataset (OR 1.02; 95% CI 1.01–1.03; *p* = 5.30 × 10⁻⁴) that did not replicate in the validation dataset (OR 1.03; 95% CI 0.87–1.21; *p* = 0.77), where the confidence interval spans the null. The manuscript therefore does not invoke genetic evidence for the reverse pathway; the pharmacological direction — medication overuse disrupting sleep architecture — is argued from clinical withdrawal data alone. Sensitivity analyses reported by the original authors (MR-Egger and weighted median) were directionally consistent with the inverse-variance-weighted estimate; heterogeneity was present in three of four datasets and was addressed with IVW random-effects models. Significant pleiotropy was detected in the finn-b-G6_MIGRAINE validation dataset (MR-Egger intercept *p* = 8.5 × 10⁻^3^), a finding that limits confidence in that particular estimate and is now stated explicitly. Three further caveats apply. First, MR instruments were built for migraine broadly defined, not for the MOH transition specifically; MOH involves analgesic overuse behavior, opioid neuroadaptation, and pharmacologically driven sleep disruption that GWAS instruments for migraine cannot model. All causal inferences in this review therefore apply to migraine risk as a proxy, not to the MOH phenotype directly, and future MR studies should use cohorts phenotyped strictly by ICHD-3 criteria for MOH rather than for chronic headache broadly. Second, the insomnia instruments derive from UK Biobank, which determined insomnia via a single self-reported touchscreen item (“Do you have trouble falling asleep at night or do you wake up in the middle of the night?”); these instruments capture broad insomnia liability rather than DSM-5- or ICSD-3-defined chronic insomnia disorder. Third, both the insomnia and migraine GWAS draw substantially on UK Biobank participants, creating sample overlap that can bias two-sample MR estimates and was not corrected for in the published analysis.

### Medication overuse as a potential contributor to sleep disruption

Each drug class overused in MOH disrupts sleep through mechanisms intrinsic to its pharmacology. Opioids suppress orexinergic neuronal firing and truncate slow-wave sleep; barbiturates and benzodiazepines fragment sleep continuity and suppress REM architecture; triptans may perturb sleep-related serotonergic pathways, though the degree and clinical significance of any sustained circadian effect with chronic use remain poorly characterized. These are not side effects in any conventional sense. They are direct consequences of engaging the same molecular targets that regulate restorative sleep.

Withdrawal studies support this direction of causation. In a randomized comparison of treatment strategies and in multiple independent prospective cohorts, patients who achieved medication cessation reported improvement in headache frequency (27); parallel improvement in self-reported sleep quality has been described after withdrawal, though it was not a pre-specified outcome in the controlled trials. The temporal sequence — overuse precedes MOH, successful withdrawal precedes sleep improvement — is consistent with MOH driving the sleep disturbance rather than the reverse, though constitutional vulnerability cannot be excluded. The critical gap, however, is that virtually every withdrawal study has measured sleep by questionnaire. Whether slow-wave architecture objectively recovers after detoxification, how long that recovery takes, and whether outcomes differ meaningfully between drug classes are questions that have not been asked with polysomnography.

## Clinical features and comorbidities

### Clinical presentation

The clinical picture when MOH and insomnia coexist is recognizable once you know to look for it. Headache is nearly constant — a dull, low-grade pressure that periodically escalates into a full attack, reliably returning the patient to the medications that sustain the cycle (4). Morning headache on waking is a near-universal complaint, mechanistically consistent with nociceptive arousal from REM sleep when monoaminergic descending inhibition is at its daily minimum. Sleep complaints are predominantly non-restorative rather than sleep-onset in character — patients wake exhausted regardless of how many hours they spent in bed, which argues against simple pain-induced wakefulness as the sole mechanism. What is perhaps most instructive, from a psychiatric vantage point, is how frequently this insomnia is present even in patients without overt mood disturbance. That persistence suggests that in at least a subset of cases, the sleep problem is not simply secondary to the pain, but reflects primary neurobiological dysregulation that will not resolve when the headache does (28).

### The COMISA phenotype

OSA is substantially more prevalent among patients with chronic migraine than in headache-free controls (29), and when OSA and insomnia coincide — the COMISA phenotype — the resulting subgroup is distinctly harder to treat. The two conditions are not additive: their co-occurrence amplifies pain sensitivity and treatment resistance through mechanisms that include recurrent trigeminovascular activation from intermittent nocturnal hypoxia (30, 31). Consider the practical implication. A patient with undiagnosed OSA who enters a detoxification program carries a nightly nociceptive stimulus that medication withdrawal cannot eliminate. Sleep medicine has documented this problem from the other direction: CBT-I efficacy is substantially reduced when apnea is untreated, and CPAP adherence drops when concurrent insomnia is ignored (31). Whether systematic screening for sleep-disordered breathing at the initial evaluation, rather than as a contingency reserved for treatment failure, improves MOH outcomes has not been tested and remains a question for prospective study.

### Psychiatric comorbidities

Comorbid anxiety is present in up to 50% of MOH patients; comorbid depression in approximately 40% (4). The biological substrate for this overlap is not obscure: HPA axis dysregulation, monoaminergic dysfunction, and limbic hyperactivation are common currency across pain processing, sleep regulation, and mood disorders. Debating whether psychiatric illness precedes or follows the pain-sleep cycle is often clinically fruitless — the answer likely varies by patient and is rarely recoverable from a single consultation. What is reliably true, and what matters for management, is that untreated anxiety or depression at baseline predicts both poorer detoxification response and higher relapse rates at one year.

## Therapeutic considerations and research hypotheses

The logic of sequential treatment — manage the headache first, then return to sleep — maps poorly onto the neurobiology described above. No randomized trial has yet directly tested a fully integrated protocol against detoxification alone, and that absence of evidence is itself a finding that should temper the claims made in this section. What follows reflects mechanistic reasoning and clinical plausibility, not a body of confirmatory controlled evidence.

### Detoxification

Stopping the overused drug remains the single most effective step for breaking the headache cycle (8). The appropriate strategy depends on drug class: opioids and barbiturates require gradual tapering to prevent dangerous withdrawal syndromes; abrupt cessation is safe and preferred for triptans and simple analgesics (32). The first two to three weeks are typically the most challenging — headaches often worsen and sleep further deteriorates before either improves — a pattern that requires proactive patient education and close monitoring. One-year relapse rates of 20%–40% underscore that detoxification is necessary but not sufficient (3, 8).

### Preventive pharmacotherapy

Some randomized and prospective studies, as well as several contemporary treatment frameworks, have evaluated preventive therapy initiated during or alongside detoxification (32). Topiramate, onabotulinumtoxinA, and the anti-CGRP monoclonal antibodies (fremanezumab, eptinezumab, and galcanezumab) have been evaluated in MOH-defined subgroups of migraine-prevention trials. Anti-CGRP agents are discussed here as a hypothesis for investigation, not a clinical recommendation. Available evidence derives from *post-hoc* analyses of trials in chronic migraine with medication overuse (including HALO CM with fremanezumab (33), PROMISE-2 with eptinezumab (34), and galcanezumab studies with MOH-defined subgroups (35); none of these trials pre-specified insomnia or sleep architecture as a primary or secondary endpoint. Whether CGRP pathway modulation secondarily benefits sleep continuity in MOH with comorbid insomnia therefore remains speculative and requires dedicated investigation. The editorial role of one co-author as co-editor of this Research Topic is disclosed; this section is presented strictly as mechanistic rationale. Whether initiating preventives before detoxification is complete confers additional benefit over detoxification alone has not been definitively resolved (32).

### Cognitive behavioral therapy for insomnia (CBT-I)

CBT-I is the guideline-recommended first-line treatment for chronic insomnia. In chronic pain populations, insomnia-focused behavioral treatment has been associated with improvements in sleep and, in some studies, pain-related outcomes (36), and this approach is clinically plausible and mechanistically coherent when CBT-I is delivered concurrently with — rather than after — detoxification, even though the early weeks are the most demanding for patients. Because insomnia in MOH is often primary rather than reactive, deferring CBT-I until headache is controlled leaves an active driver of central sensitization untreated throughout the most critical phase of recovery. For patients with the COMISA phenotype, CBT-I is best delivered in parallel with CPAP when possible; available evidence suggests that addressing either disorder without the other tends to yield incomplete responses (31). Whether adding concurrent CBT-I to standard detoxification improves durable remission rates remains to be established in a randomized controlled trial.

### Dual orexin receptor antagonists (DORAs)

Daridorexant, suvorexant, and lemborexant block both OX1R and OX2R. Unlike benzodiazepines and z-drugs, they do so without suppressing SWS or REM — preserving the sleep stages most protective against pain sensitization. Their mechanism directly targets the orexinergic system implicated in MOH — a pathway plausibly involved in the sleep and pain dysregulation seen in this population. No dedicated trials have been conducted in MOH with comorbid insomnia. The mechanistic rationale for DORAs in this context requires restatement in light of the CSF data: in a system where orexin-A is elevated rather than depleted, receptor antagonism could dampen an overdriven signal — but if the elevation reflects a compensatory antinociceptive drive, receptor blockade could in principle worsen pain. This unresolved direction of effect is a specific reason to treat DORAs as a hypothesis requiring prospective evaluation with prespecified bidirectional outcomes (sleep architecture and headache frequency) before any clinical guidance can be offered. DORAs are presented here not as a clinical recommendation but as a mechanistically plausible, directionally uncertain candidate for phase II investigation; the editorial role of one co-author as co-editor of this Research Topic is disclosed in this context.

### A note on other pharmacological sleep aids

Benzodiazepines and opioid-based hypnotics should generally be avoided in this context: both suppress SWS and REM and carry substantial risk of inducing or perpetuating MOH. Melatonin at 3 mg at bedtime may warrant evaluation as a low-risk adjunct offering circadian stabilization, though its analgesic value in MOH specifically is untested (18).

## Discussion

The persistence of this clinical problem is not explained by the co-occurrence of two common conditions. It is explained by the architecture of their interaction. Orexinergic dysregulation, melatonin blunting, neuroinflammation, and altered thalamocortical sensory processing (a construct derived primarily from preclinical models of chronic pain and non-MOH-specific human imaging studies, not from MOH-dedicated investigations) — described here as impaired modulation of nociceptive input at the thalamic level during sleep — are mechanistically coupled in preclinical data and indirect human evidence. Perturbing any one of them tends to drive disruption in the others, and direct MOH-specific data remain limited. Detoxification pursued without concurrent sleep treatment leaves the insomnia-driven component of central sensitization intact at exactly the moment when recovery is most vulnerable. The reverse failure mode is equally real: attempting behavioral sleep intervention while analgesic overuse continues ensures the pharmacological disruption of sleep architecture will neutralize whatever CBT-I might otherwise accomplish.

At the interface of headache medicine and psychiatry, the sleep problem tends to be addressed as an afterthought, if it is addressed at all. This is a strategic error that shows up consistently in the relapse data. Patients who complete detoxification successfully but return to poor sleep are not failures of will or of the detoxification protocol; they are patients whose neurobiological substrate was left intact. Orexinergic dysregulation, melatonin blunting, and glial inflammation do not necessarily resolve with analgesic cessation, and there is no reason to expect that they would. Waiting to address them ensures a meaningful proportion of those patients will reinitiate analgesics at the next sufficiently painful episode.

Several tensions in the evidence deserve acknowledgment. Mendelian randomization strengthens the inference that insomnia liability contributes to migraine risk, but the instruments were built for migraine broadly, and the MOH phenotype — involving behavioral reinforcement, opioid neuroadaptation, and additional layers of central sensitization — may not be captured by those same genetic instruments. Whether concurrent management is preferable to sequential treatment remains an open clinical question; no randomized trial has compared integrated treatment against detoxification alone, and the mechanistic argument should not substitute for that evidence. The COMISA phenotype is perhaps the most underappreciated gap: well characterized in general sleep medicine populations, its specific prevalence, clinical predictors, and treatment response in MOH cohorts have not been systematically studied.

From a clinical standpoint, two changes in practice appear most immediately actionable. Sleep-disordered breathing screening may be particularly valuable at the initial consultation in headache centers, especially in high-risk patients, rather than deferred until standard treatment fails. A patient with undiagnosed OSA who undergoes detoxification faces a persistent nightly nociceptive stimulus that analgesic withdrawal alone may not neutralize. Whether brief screening for sleep-disordered breathing alters management in this setting has not been established. The second change is conceptual: insomnia in MOH is not an afterthought to be addressed after headache control. It appears to be part of the mechanism sustaining the disorder, and addressing it early in the treatment plan is mechanistically coherent.

This review has limitations inherent to the narrative format: no pre-registered protocol, a broad but non-exhaustive search, evidence appraised narratively rather than through GRADE, and no formal meta-analysis. These limitations point toward specific research priorities. Polysomnographic studies tracking sleep-architecture recovery longitudinally through detoxification — disaggregated by drug class — are an important priority for the field. A randomized trial of concurrent CBT-I plus detoxification versus detoxification alone is the highest-priority clinical investigation in the field. Phase II trials of DORAs in MOH with comorbid insomnia are mechanistically justified and warrant prospective investigation. Mendelian randomization analyses using instruments specific to the MOH phenotype would sharpen causal inference considerably. Polysomnographic outcomes should be pre-specified endpoints in all future detoxification trials, not optional additions.

## Conclusion

The co-occurrence of MOH and insomnia is not coincidental, and it is not simply a matter of shared risk factors. These conditions appear to sustain one another through overlapping neurobiological substrates — orexinergic dysregulation, melatonin blunting, circadian dysregulation, glial neuroinflammation — each of which amplifies both disorders simultaneously. It bears repeating that direct causal evidence here pertains to migraine broadly, not to the MOH phenotype specifically; the MOH transition adds behavioral and pharmacological dimensions that existing genetic instruments do not capture. Psychiatric comorbidity and the COMISA phenotype compound the picture further, each adding independent layers of treatment resistance.

Sequential management fails a predictable subset of these patients because it leaves central sensitization fueled by unaddressed sleep pathology throughout the phase when recovery is most fragile. The mechanistic evidence is consistent with the hypothesis that an integrated approach — concurrent detoxification, insomnia-focused treatment, and preventive pharmacotherapy introduced in parallel, not in series — may yield better long-term outcomes than sequential management, though this hypothesis remains untested in a randomized controlled trial. Anti-CGRP agents and dual orexin receptor antagonists represent mechanistically plausible hypotheses for future investigation at the intersection of both disorders; neither should be presented, at this stage of evidence, as a clinical recommendation.

Insomnia in MOH is not a symptom waiting passively for the headache to resolve. It appears to be a sustaining mechanism in its own right — one that perpetuates central sensitization, degrades the neurobiological conditions for recovery, and reliably predicts relapse when left unaddressed. Treating it from the outset of the therapeutic plan, rather than deferring it, may prove to be what distinguishes durable remission from repeated cycles of detoxification and relapse. The absence of randomized evidence for integrated management, now clearly acknowledged, is not a reason to defer clinical thinking but a research agenda. The trial needed is defined: concurrent withdrawal, CBT-I, and preventive pharmacotherapy versus detoxification alone, with polysomnographic sleep architecture and one-year relapse as co-primary endpoints. The orexinergic paradox identified in CSF data and the unresolved mechanism of CGRP-pathway effects on sleep continuity represent two additional translational questions that phase II investigations could address simultaneously.
