# Supplementary material for: Insomnia and medication overuse headache: neurobiological links, reciprocal interactions, and integrated therapeutic strategies
Source: Front Pain Res (Lausanne). 2026 Oct 16;7:1911261. doi: 10.3389/fpain.2026.1911261 (PMC13720594; doi:10.3389/fpain.2026.1911261)
Supplement: Supplementary file 1 [file Table1.docx]

**Supplementary Material**

*Insomnia and Medication Overuse Headache: Neurobiological Links, Reciprocal Interactions, and Integrated Therapeutic Strategies*

Frontiers in Pain Research · Manuscript ID 1911261

**Supplementary Methods 1 — Full search strategy**

This narrative review followed a structured, reproducible search. It was not registered as a systematic review, and no formal protocol was pre-specified. The complete strategy, per-database yields, and deduplication path are reported below so that the search can be independently reproduced.

**1. Databases, dates, and per-database yields**

**Databases:** PubMed (MEDLINE) and Embase.

**Coverage:** Database inception to 20 June 2026.

**Search executed:** 16 July 2026.

**PubMed:** 207 records (primary string, 87; supplementary string, 120).

**Embase:** 670 records (primary string, 196; supplementary string, 474).

**Total exported (raw):** 877 records.

**Supplementary source:** Google Scholar was used only for supplementary citation tracking (first 100 results per supplementary search, sorted by relevance) to identify highly cited articles not indexed in PubMed or Embase; these records were evaluated separately and did NOT contribute to the deduplicated pool.

**2. Deduplication and screening path**

877 records were exported and deduplicated, yielding 730 unique records. These were screened by title and abstract against the eligibility criteria; records judged potentially relevant were examined in full text, and those meeting the criteria were retained for the narrative synthesis. The 36 sources cited in this review constitute its evidence base.

Deduplication was performed by matching on normalized title and DOI. Records sharing a container DOI (for example, conference-supplement abstracts) and generically titled records were disambiguated by title-plus-year rather than DOI alone to avoid false-positive merges.

**3. Boolean search strings**

**Primary string (PubMed and Embase; MeSH and free-text combinations):**

("medication overuse headache" OR "analgesic rebound headache") AND ("sleep disorders" OR "insomnia" OR "sleep apnea" OR "orexin" OR "hypocretin" OR "melatonin" OR "circadian")

**Supplementary string (mechanistic and therapeutic evidence; not used to estimate insomnia prevalence in MOH):**

("Mendelian randomization" OR "CBT-I" OR "dual orexin receptor antagonist" OR "CGRP") AND ("headache" OR "migraine") AND ("sleep" OR "insomnia")

**4. Eligibility criteria**

**Included:** English-language, peer-reviewed original clinical studies, observational cohorts, genetic analyses, mechanistic studies in humans or validated animal models, randomized trials in insomnia or migraine populations, and major reviews directly relevant to MOH, insomnia, orexin biology, circadian rhythms, CBT-I, dual orexin receptor antagonists, or anti-CGRP therapies.

**Excluded:** Editorials, commentaries, and letters; conference abstracts and other non-peer-reviewed meeting material; book chapters; preprints (including bioRxiv and medRxiv); case reports unless mechanistically informative; non-English publications; and duplicate or superseded reports.

**Selection:** Source selection was carried out by the first author and reviewed by the senior author. No systematic forward or backward citation chasing was performed, and trial registries (ClinicalTrials.gov, WHO ICTRP) were not searched; these are acknowledged as limitations.

**5. Evidence appraisal**

Randomized controlled trials were assessed for allocation concealment and blinding; observational studies were appraised for selection bias and confounding. A formal certainty-grading framework (e.g., GRADE) was not applied, because GRADE rates certainty across a body of trials addressing a defined comparative question, and no body of trials comparing integrated against sequential management of MOH with comorbid insomnia exists — an absence that is itself a central observation of this review.
